# Long term results of permanent epicardial pacing in neonates and infants

**DOI:** 10.1186/1749-8090-10-S1-A207

**Published:** 2015-12-16

**Authors:** Sungkyu Cho, Woong-Han Kim, Kwanyong Hyun, Ji Young Park, Jeong Ryul Lee, Yong Jin Kim

**Affiliations:** 1 Seoul National University Children's Hospital Department of Thoracic and Cardiovascular Surgery, Seoul National University College of Medicine Seoul National University Hospital, Seoul National University Children's Hospital 101 Daehangno, Jongno-Gu, Seoul, 110-744, Korea

## Background/Introduction

Despite the improvement of transvenous leads, the open surgical implantation of epicardial leads in neonates and infants remains essential.

## Aims/Objectives

We reviewed the long term outcomes after epicardial pacemaker implantation in neonates and infants.

## Method

From 1989 to 2013, 44 patients (15 neonates) underwent pacemaker implantation within the first year of life. The mean age and weight were 101.2 ± 97.2 days and 4.8 ± 1.8kg at the time of pacemaker implantation. The Indication for pacemaker implantation were postoperative atrioventricular block in 23, congenital atrioventricular block in 16, sinus node dysfunction in 4, and myocarditis induced atrioventricular block in 1. Unipolar epicardial leads (non steroid eluting: 22, steroid eluting: 22) were inserted through median sternotomy or subxyphoid approach.

## Results

Mean follow up duration was 9.1 ± 7.9 years. The most commonly used generator mode at first implantation was VVI, which was used in 52.3% (n = 23). There were generator mode changes from initial VVI or VVIR to dual chamber pacing in 11 patients, mean 7.0 ± 6.2 years later, due to ventricular dysfunction and dyssynchrony. Freedom from the need for generator replacement was 95.0%, %, 70.1%, 21.7% at 1, 5, 10 years. And, the median longevity of the generators was 6.5 years. 15 lead malfunction (34.1%) events were detected, 5 of which were due to lead fracture and 1 of which was due to lead infection. Freedom from the need for lead replacement was 97.6%, 75.7%, 47.7% at 1, 5, 10 years. The lead replacement rate is significantly higher in patients who underwent non steroid eluting lead implantation (p = 0.033). The median longevity of the leads was 9.1 years.

## Discussion/Conclusion

In neonates and infants, more frequent pacemaker generator or lead changes are needed because of high pacing rates, complete pacemaker dependency and rapid somatic growth. The use of steroid eluting leads increased lead longevity and decreased the need for surgical reinterventions. Epicardial pacing is feasible, safe, and effective in neonates and infants.

